# Seven Common Surgical Follies

**Published:** 1884-04-20

**Authors:** John B. Roberts

**Affiliations:** Surgeon to St. Mary’s Hospital


### SEVEN COMMON SURGICAL FOLLIES.

BY JOHN B. ROBERTS, M.D., SURGEON TO ST. MARY'S HOSPITAL.

**Read before the West Chester Medical Society, February 13, 1884.**

In complying with the request of your committee, I shall offer, this evening, some criticisms on a number of prevalent surgical methods and opinions which I believe to be fallacious. If I cast and trample in the dust any idols dear to the hearts of my hearers, I trust they will receive my iconoclastic doctrines with the thought that my own folly has, in many cases, been my teacher, and that I do not attempt to force conviction upon the members of this Society with any other weapon than words.

If any one of you will watch with careful scrutiny any series of operations done for various lesions and by various surgeons, you will have frequent opportunity of observing the commission of the seven follies that I shall describe. Sometimes you will see a single operator committing nearly every one of them in as many minutes. I call them the ether folly, the incision folly, the sponge folly, the styptic folly, the suture folly, the adhesive plaster folly and the dose folly.

The ether folly is almost universal. Often have I heard physicians say of a patient, "He couldn't be etherized; I had to give chloroform." Now, the fault was not with the patient, but with the doctor. I doubt there being an individual or animal in the world that cannot be anæsthetized with ether properly administered. It must, however, be given in large quantity and with little air. If given in small quantity and with much air, as chloroform should be administered, the excitement stage will only be overcome with much difficulty and loss of time. When the napkin saturated with ether has once been placed over the patient's nose and mouth it should not be removed. As it becomes necessary to replenish the anæsthetic. let the etherizer turn up the corner of the ether napkin and quickly dash upon it a fluidounce of the anæsthetic; or let him pour it on the outside of the napkin, and cover this with a large, dry towel. To remove the napkin entirely from the face while the stoppel is being taken from the bottle and the ether slowly poured out is too ridiculous for credence; yet it is the usual method. During this interval the patient takes two or three inhalations of pure air, and thus neutralizes the effect of most of the ether previously inhaled. There is one symptom, however, that demands removal of the ether towel for a moment: it is the blue and congested face, due to spasm of the respiratory muscles, that often occurs soon after the commencement of etherization. When this is seen the patient should be given an opportunity to take *one* deep inspiration of air· The towel should then be immediately replaced. A tendency to retch does not indicate cessation, but continuance, of etherization, since a fully etherized patient never vomits. If food actually comes up into the fauces, the patient must be given a chance to expel it, lest a particle get into the larynx. This, however, takes but a moment, after which the ether must be quickly resumed. If the trachea becomes full of rattling mucus, the patient should be turned on his abdomen with the head dependent and the ether, perhaps, removed from his face for a moment until the mucus has an opportunity to escape from the mouth. I do not advocate giving ether carelessly, but I assert that it is usually given inefficiently. More danger is to be found in this long-continued inefficient etherization than in the prompt method I describe. To gain the patient's confidence, I get him to breathe deeply with his face covered with a dry towel for about a minute before pouring on the anæsthetic. Squibb's ether is in no way superior to that of other reputable manufacturers.

The incision folly is not quite as common as the one just discussed. Still it is often exhibited in both hospital and private operating. It consists in making a cramped cutaneous incision, instead of one sufficiently large to fully display the tissues needing examination. A cut of the skin three inches long is no more dangerous in itself than one two inches long. Indeed, in many cases it is less so, because the surgeon, having sufficient room to see, does not tear and stretch the underlying tissues so rudely; hence, less suppuration occurs and more rapid union is possible. In opening abscesses, as in general operating, a free cut is more satisfactory to the surgeon and more beneficial to the patient than a mere puncture or button-hole incision. Let us keep from this folly, then, by using a keen edge and a free hand in making cutaneous operation wounds.

What I term the sponge folly is the habit of employing sponges for absorbing blood from wounds, when napkins or towels are always obtainable, and are far less liable to introduce septic material into the wound. Sponges, while too expensive to be thrown away after each operation, are cleaned with great difficulty. Servants and nurses, therefore, not appreciating the importance of thorough cleansing and disinfection, often neglect this duty. Hence, I prefer towels, and if I do an operation at a patient's house, always use clean towels obtained there. Thus I secure an almost certain immunity from purulent or septic dirt in the articles used for absorbing blood. Perfectly clean surgical sponges are the exception, but clean household towels are the rule. At the Polyclinic I use, for this purpose, to a considerable extent, Japanese paper napkins, which are thrown away after being once used. Absorbent cotton is too expensive for such uses, except to a limited extent, and besides has a tendency to leave filaments entangled in the wound.

The styptic folly is the commonest and most ridiculous of the surgical traditions of the present day. When occlusion of each bleedlng vessel by ligation, torsion or acupressure is not required (and it seldom is for arteries smaller than the facial), moderate direct pressure is all that is demanded. Styptics should not be used, because not needed, and because, in many instances, they impede union of the wound. After an operation let the surgeon tie the large vessels, wipe away the clots, put in the sutures, apply moderate equable pressure by compress and bandage, and he will have no need of hot water, alum, tannin or that vilest of all styptics, Monsel's solution.

The objections to styptics are these:

Their traditional reputation leads to their use when ligation, torsion or acupressure is needed. If they fail to arrest the bleeding, valuable time has been lost, and the pasty clots often formed by their use render isolation and ligation of the vessels difficult. Many styptics, though not all, delay union by irritating the cut surfaces and inducing suppuration.

In my hospital and college work, pressure and ligatures are the only hæmostatic agents I require. Indeed, pressure will often take the place of many ligatures. I recently excised an entire breast, with part of the great pectoral muscle, and only needed three or four ligatures. I have often, however, seen many unnecessary ligatures applied in such cases. In these days of absorbable ligatures, the practice is not as objectionable as in former years, when many long strings, to act as setons, were left hanging out of the wound.

The suture folly next claims attention. I do not refer to the erroneous opinion, long held, that sutures should not be used in the scalp. This tradition has been disproved so often that few surgeons would now hesitate to use sutures as freely in the scalp as elsewhere. What I call the suture folly is the adherence of many to the theory that silver wire only should be employed for suturing purposes. Nothing could be more fallacious. Do we use silver hare-lip or acupressure pins? Why, then, employ silver sutures, when iron wire is stronger and far cheaper? When large and gaping wounds require the sutures to stand much tension, silver wire, if used, must be very thick. Iron wire of much smaller diameter, and therefore much more flexible, gives an equally strong suture, and in addition to being better adapted to the purpose, is much cheaper. I recollect that, in hospital practice, nearly eight years ago, I discarded silver wire, which cost one dollar for each small coil, and bought, at a hardware store, enough iron wire for ten or fifteen cents to last many months. The nicest iron wire I have seen, and which I now use for the purpose, because it is strong, very flexible and free from elasticity, can be bought for five cents a spool. If it becomes a little rusty, it can be rubbed clean in a moment, should the operator object to the small amount of oxide of iron upon it.

The adhesive plaster folly is common. You all have seen stumps, after amputation, enveloped more or less completely in strips of adhesive plaster placed between the sutures. Of what use are they? They obstruct free drainage, become softened and loosened by the pus, if there is much discharge, give more or less pain when removed, and do no good. If the flaps are properly made, the sutures correctly applied and the stump neatly and evenly bandaged, the adhesive plaster becomes useless, and is merely a disadvantage to the patient's comfort and recovery. Adhesive plaster has little or no value in surgery, except for making extension and preventing motion in cases of fracture.

I believe that operative surgery will be greatly improved as a scientific entity when sponges, styptics, silver wire and adhesive plaster are discarded in the dressing of wounds. If you have these articles for this purpose in your offices, I pray you to throw them away. They are needless, worthless and detrimental. It is our natural adherence to what is traditional that impedes progress in this, as in other branches of scientific learning. We need, indeed, a Leo and a Constantine to destroy these valueless relics of ancient surgical worship, as we need an Alexandrian fire to consume the thousands of worthless splints and instruments that are still described in surgical text-books to the confusion of the student and the damage of the community.

The dose folly is the last topic I shall discuss with you this evening. I should, perhaps, term it the *small* dose folly, for I refer to the practice of administering insufficient doses of medicines. This fault pertains, of course, to medical as well as to surgical practice. Nearly every year of my professional life leads me to increase the dose of some one or other of the articles that I am accustomed to use. Of what use is a sixteenth or an eighth of a grain of morphia to a man with severe pain? Give him a quarter of a grain or even a half, repeated if necessary, and he will soon be comfortable and thankful. Perhaps he will also pay his bill. The medical requirements of to-day are drugs and doses with inherent power. You can't lift a block of granite with a weak crowbar; neither can you cure agony with a debilitated dose of anodyne. So it is with all other remedies. If any medication at all is required, give that which will do the work and do it promptly. A few large doses will dispel the symptoms and cure the patient, when months of nonsensical drugging with emasculated remedies will bring nothing but discredit to the practitioner and obloquy to medical science. I have spoken of morphia as a type, but the same remarks hold good concerning quinia, atropia, strychnia, digitalis, iodide and bromide of potassium, mercury, pilocarpia and, indeed, of all our remedies. Use the alkaloids or active principles in every case. Then you will know what you are giving, and you will soon learn that much larger doses are tolerated than is usually thought possihle. Many physicians and surgeons fail to cure; not because of faulty diagnosis, not because of inappropriate remedies, but because of insufficient dosage.

I have, to-night, Mr. President, endeavored to convince the members of this society that much that is believed and practiced in surgery is false; that its only virtues are its hoary antiquity and its very general acceptance. I do not, however, condemn these opinions and methods because they are old, but because I believe them to be wrong. Old age is, indeed, to be revered, but its errors are none the less egregious. Devotion to traditional ideas has delayed the progress of scientific surgery very many decades. Painstakiug investigation, accurate anatomical knowledge and common sense have uncovered and dispelled the folly of many of these surgical superstitions; and the seven follies just described, with many others that I might have discussed, will probably soon have few believers and followers. Let us hope that in small, as well as in great matters, surgical sense will soon take the place of surgical nonsense.—*Polyclinic.*

